# The Innate Immune Signalling Pathways: Turning RIG-I Sensor Activation against Cancer

**DOI:** 10.3390/cancers12113158

**Published:** 2020-10-27

**Authors:** Sandra Iurescia, Daniela Fioretti, Monica Rinaldi

**Affiliations:** Institute of Translational Pharmacology (IFT), Department of Biomedical Science, National Research Council (CNR), 00133 Rome, Italy; daniela.fioretti@ift.cnr.it

**Keywords:** innate immunity, RIG-I, agonist, tumour microenvironment, cancer, immunotherapy, clinical trial

## Abstract

**Simple Summary:**

The clinical success in immunotherapy has been remarkable, and, at the same time, disappointing. The immune context of the tumour microenvironment has an influence on tumour initiation, response, and therapy. It is an urgent matter to explore mechanisms shaping the tumour microenvironment for further progression of immunotherapy. The immunoreceptor retinoic acid-induced gene-I (RIG-I) has emerged as a promising target molecule to activate adoptive immunity via activation of innate immunity. In this paper, we highlight basic mechanisms of RIG-I signalling in the tumour microenvironment, broadening to the most recent preclinical studies that employ RIG-I agonists. We also present an up-to-date selection of clinical trials designed to prove the antitumour role of RIG I, and that may result in improved therapeutic outcomes for cancer patients.

**Abstract:**

Over the last 15 years, the ability to harness a patient’s own immune system has led to significant progress in cancer therapy. For instance, immunotherapeutic strategies, including checkpoint inhibitors or adoptive cell therapy using chimeric antigen receptor T-cell (CAR-T), are specifically aimed at enhancing adaptive anti-tumour immunity. Several research groups demonstrated that adaptive anti-tumour immunity is highly sustained by innate immune responses. Host innate immunity provides the first line of defence and mediates recognition of danger signals through pattern recognition receptors (PRRs), such as cytosolic sensors of pathogen-associated molecular patterns (PAMPs) and damage-associated molecular pattern (DAMP) signals. The retinoic acid-inducible gene I (RIG-I) is a cytosolic RNA helicase, which detects viral double-strand RNA and, once activated, triggers signalling pathways, converging on the production of type I interferons, proinflammatory cytokines, and programmed cell death. Approaches aimed at activating RIG-I within cancers are being explored as novel therapeutic treatments to generate an inflammatory tumour microenvironment and to facilitate cytotoxic T-cell cross-priming and infiltration. Here, we provide an overview of studies regarding the role of RIG-I signalling in the tumour microenvironment, and the most recent preclinical studies that employ RIG-I agonists. Lastly, we present a selection of clinical trials designed to prove the antitumour role of RIG I and that may result in improved therapeutic outcomes for cancer patients.

## 1. Introduction

The tumour microenvironment (TME) and the associated inflammatory processes play a significant role in cancer progression. The role of the immune cells in shaping the TME has been unequivocally established.

Consequently, tumour subtypes can be classified as immune desert, excluded or inflamed, depending on density and diversity of tumour-infiltrating immune cells [1,2,3,4,5].

Neoplastic cells develop various strategies to evade the host immunosurveillance [6,7]. In particular, they are able to modulate intrinsic (i.e., immunosuppressive cytokines and cell types) and extrinsic pathways (i.e., T-cells exhaustion) to elude endogenous anti-tumour immunity, to establish an immunosuppressive TME, and to foster cancer progression [8]. In the immunosuppressive TME, particularly in T-cell inflamed tumours, several types of tumour-promoting cells are present, such as CD4^+^ regulatory FoxP3^+^ T-cells, tumour-associated macrophages, myeloid-derived suppressor cells, and infiltrating cytotoxic CD8^+^ T-cells. However, many CD8^+^ T-lymphocytes show an anergic phenotype and their function is impaired [9].

Advances in understanding the relationship between cancer and the immune system have enabled the development of several immunotherapeutic approaches aimed at targeting the mechanism of immune evasion to fight cancer.

Immune checkpoint blockade (ICB), a therapeutic approach that inhibits negative regulatory immune checkpoints, has led to significant progress in cancer therapy. Nevertheless, immunological composition and functional status of the TME can affect responsiveness to ICB therapy. Preclinical studies targeting cytotoxic T-lymphocyte-associated protein-4 (CTLA-4), programmed death-ligand 1 (PD-L1), and indolamine 2,3-dioxygenase (IDO) have shown that therapeutic effects are associated with re-activation of CD8^+^ tumour-infiltrating lymphocytes (TILs) within the TME [10].

Consistent with these data, immune checkpoint inhibitor (ICI) treatments have shown clinical benefits for a minority of patients, whose tumours are immunogenic and T-cell inflamed. In contrast, ICI monotherapy is less effective in TME that are devoid of T-cells and infiltrated by immunosuppressive cells [1,9].

A relevant feature of T-cell inflamed tumours is the expression of type I interferons (IFNs) and IFN-inducible genes [11].

The notion that type I IFN signalling contributes to spontaneous activation of anti-tumour T-cell response has recently emerged, highlighting the correlation between innate and adaptive responses [12,13]. Such evidence has prompted the exploration of new therapeutic strategies aimed at improving tumour immunogenicity. The activation of the innate immunity is a promising approach to promote de novo inflammation in the TME and that could contribute to overcome its critical suppressive barrier, skewing non-immunogenic into immunogenic tumours [14,15].

One emerging strategy exploits the potential of cytosolic sensors belonging to pattern recognition receptors, whose roles are the detection of pathogen-associated molecular patterns (PAMPs) and damage-associate molecular pattern (DAMP) signals. Nucleic acid-sensing, an essential mechanism of the innate immunity, utilises cytosolic receptors to detect extranuclear DNA or extracellular RNA as DAMPs and PAMPs [16]. In mammalian cells, two paradigmatic pathways are activated by the cyclic GMP-AMP synthase (cGAS)/stimulator of interferon genes (STING), which is responsible for cytosolic DNA sensing, and the retinoic acid-induced gene-I (RIG-I)-like receptors/mitochondrial antiviral-signalling (MAVS) protein, which detects cytosolic RNAs. Once engaged, these nucleic acid-sensors activate multiple signalling pathways that converge on the production of type I IFNs and pro-inflammatory cytokines, facilitating T-cell cross-priming and infiltration [17,18].

In the tumour context, the cGAS/STING pathway appears to be the major innate immune DNA sensing pathway that drives dendritic cells (DCs) activation and subsequent T-cell priming against tumour-associated antigens in vivo [11]. Therefore, STING agonists are being investigated as cancer therapeutics to activate or mimic the cGAS/STING signalling pathway for promoting immune-mediated tumour rejection [17,19,20]. Unfortunately, clinical results obtained with STING agonists such as ADU-S100/MIW815 (NCT02675439), MK-1454 (NCT03010176), and E7766(NCT04144140 are modest and limited to patients with accessible solid tumours amenable to intratumoural delivery [19,20,21,22]. Moreover, emerging evidence suggested the pro-tumour roles of the cGAS-STING pathway in cancer progression [23,24,25], thus, limiting the application of STING agonists in the clinic.

Besides driving a pro-inflammatory transcriptional program, RIG-I signalling is able to induce the death of virally infected cells [26]. In the cancer setting, RIG-I triggers dual effects (i.e., selective tumoricidal activity and activation of immune cells in the TME) opening new therapeutic opportunities for cancer immunotherapy [26,27]. RIG-I mimetics are thus being explored as attractive agents for clinical translation to stimulate de novo inflammation, DCs activation and T-cell priming, particularly in poorly immunogenic, non-T-cell inflamed tumours.

Here, we provide an overview of studies relating to RIG-I signalling in the TME, broadening to the most recent preclinical studies that employ RIG-I agonists. Lastly, we present a selection of clinical trials designed to prove the antitumour role of RIG I and that may result in improved therapeutic outcomes for cancer patients.

## 2. RNA Recognition and Signal Activation by RIG-I Sensor 

The viral nucleic acid sensor RIG-I, encoded by the DDX58 gene (GenBank: AF038963), belongs to the DExD/H box RNA helicases and is one of three members of the protein family of RIG-I-like receptors [28]. The two other members of this family, namely melanoma differentiation associated (MDA)-5 and laboratory of genetics and physiology (LGP)-2, share with RIG-I a central helicase domain and a carboxy-terminal domain. These two domains cooperate to detect RNA molecules. RIG-I and MDA-5 harbour two amino-terminal caspase activation and recruitment domains (CARDs), devoted to downstream signal transduction, while LGP2 is lacking these tandem CARDs [29,30]. Several studies suggest that LGP2 activation affect RIG-I and MDA-5 signalling in a positive and negative manner (reviewed in [31]). A comprehensive comparative analysis of structures and functions of RIG-I and MDA-5 has been recently covered elsewhere [32].

RIG-I becomes activated upon viral RNA binding to its helicase domain and activates downstream signalling [29]. The properties of RNA molecules that are recognised by RIG-I and the activation mechanism have been extensively defined. In addition to ligand length [33,34], RIG-I engagement requires that immunostimulatory RNAs possess salient molecular and structural features [30,35,36]. RIG-I binds to short, uncapped blunt double-strand (ds) RNA duplex molecules bearing a triphosphate (3P) [37,38] and diphosphate (2P) groups at their 5′ end [39] or to RNA containing self-complementary 3′ and 5′ ends, which pair to form a secondary structure called “pan-handles” [37]. In addition, the methylation status of RNA ligands impacts on RIG-I stimulation: the 5′-terminal nucleotide needs to be unmethylated at its 2′-O position [40].

**Figure 1:**
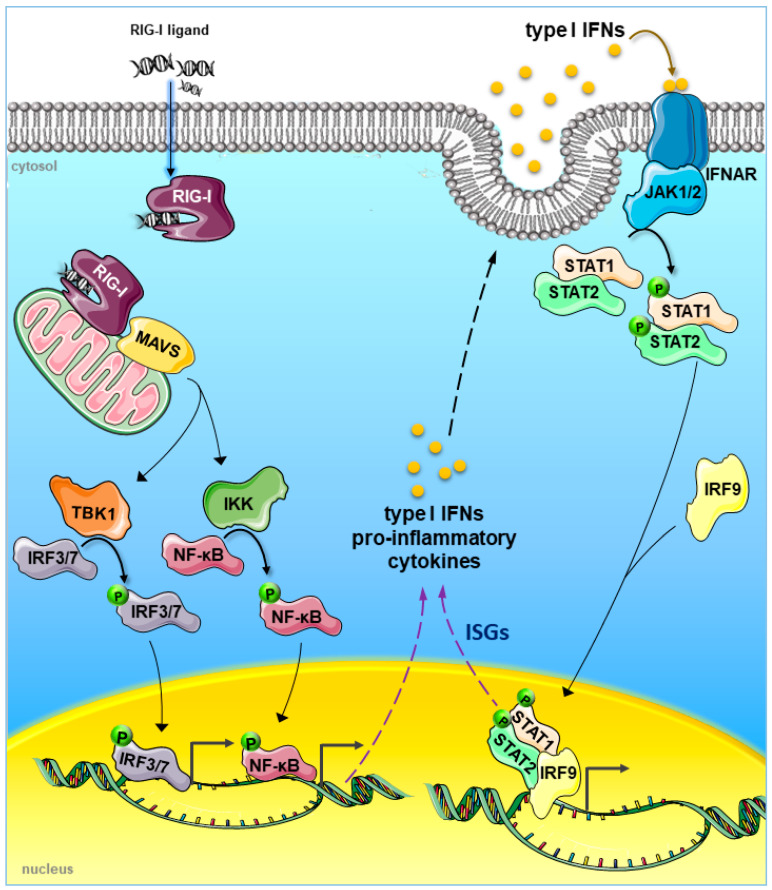
RNA ligand recognition and signal activation by retinoic acid-induced gene-I (RIG-I) sensor. Following the interaction with the ligand, RIG-I can bind to the MAVS adapter protein, anchored in the mitochondrial membrane, which recruits signalling adaptors and kinases, including the IKK family of kinases and TBK1. The signal proceeds from the activated kinases to IRF3, IRF7 and NF-κB that are in turn activated and translocated into the nucleus where they coordinate the induction of type I IFNs and proinflammatory cytokines. The binding of type I IFNs to their cognate receptor (IFNAR) leads to the transcriptional activation of ISGs by binding of ISGF3 complex containing signal transducer and activator of transcription (STAT)1, STAT2, and IRF9 to IFN-stimulated response elements (ISRE) in the promoters of ISGs. Such an ISG activation induces an amplification of the IFN-inducible feed-forward signalling axis. RIG-I, retinoic acid induced gene 1; MAVS, mitochondrial antiviral-signalling protein; IKK (or IκB kinase), inhibitor of nuclear factor kappa B; TBK, TANK-binding kinase 1; IRF, interferon regulatory factor; NF-κB, nuclear factor kappa-light-chain-enhancer of activated B cells; IFNAR, type I interferon receptor; IFN, interferon; ISGs, IFN-stimulated genes; STAT, signal transducer and activator of transcription.

Upon ligand binding, RIG-I undergoes conformational changes that render their CARDs available for interaction with the adaptor protein MAVS anchored into mitochondrial membrane, mitochondrial-associated membranes and peroxisomes. MAVS recruits signalling adaptors and kinases, including the IκB kinase (IKK) complex, and TANK-binding kinase 1 (TBK1). Briefly, a signal proceeds from the activated RIG-I to interferon regulatory factor 3 (IRF3), IRF7 and the transcription factor nuclear factor-κB (NF-κB) that are activated and translocated into the nucleus. These events culminate in the transcriptional activation of type I IFNs, proinflammatory cytokines and IFN-stimulated genes (ISGs) via a feed-forward signalling cascade [29] (Figure 1).

## 3. RIG-I Signalling Triggers Cancer Cells Death

The role of RIG-I is actually far beyond that of a pattern recognition receptor; therefore, RIG-I can be defined as a multifunctional protein [41].

RIG-I is a cytoplasmic immunoreceptor ubiquitously expressed in human tissues, and its presence across cancer cells is also well documented. A number of studies suggested that RIG-I acts as a tumour suppressor in hepatocellular carcinoma (HCC), acute myeloid leukaemia (AML), and cervical cancer [42,43,44,45,46,47].

In cancer cells, the two major outcomes of the RIG-I signalling pathway are type I IFNs production and preferential activation of programmed tumour apoptosis [48], with features of immunogenic cell death (ICD) [49,50,51].

**Figure 2:**
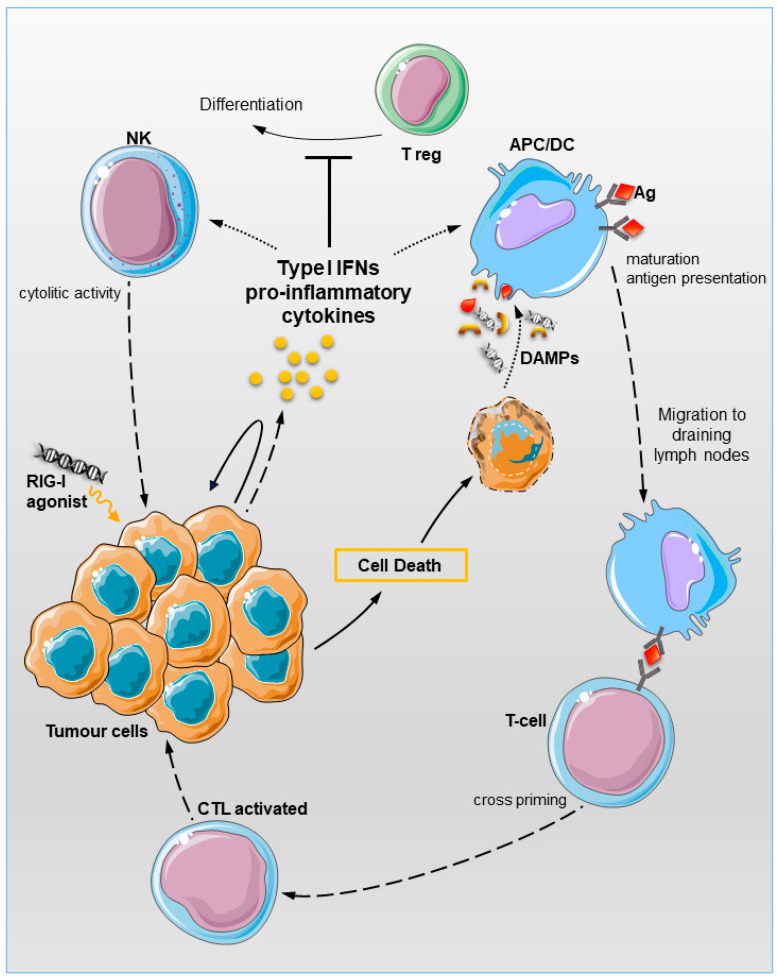
Working model of RIG-I pathway activation in the tumour microenvironment (TME). Activation of RIG-I signalling pathway in the tumour cells leads to type I IFN-driven immune response and preferential activation of programmed tumour cell death. Both the cytolytic activity of natural killer (NK) cells and phagocytic potential of macrophages are increased, while the T-regulatory cell differentiation was reduced in response to type I IFN-enriched microenvironment. Maturation of DCs will result in enhanced CD8^+^ T-lymphocytes cross-priming in tumour draining lymph nodes. RIG-I, retinoic acid induced gene 1; IFN, interferon; NK, natural killer; DC, dendritic cell; TME, tumour microenvironment.

The immunogenicity of cell death is dependent on specific pro-inflammatory signalling events triggered in dying cells. Such events include the induction of ICD-related DAMPs (release of nuclear protein high-mobility group box 1 (HMGB1) and ATP, calreticulin exposure on the outer cell membrane, as well as type I IFNs and chemokines release [52]. The different forms of ICD and DAMP signalling can orchestrate adaptive immunity against cancer cells, enabling T-cell-driven immune responses specific for antigens derived from the dying cell [52,53] (Figure 2).

**Figure 3:**
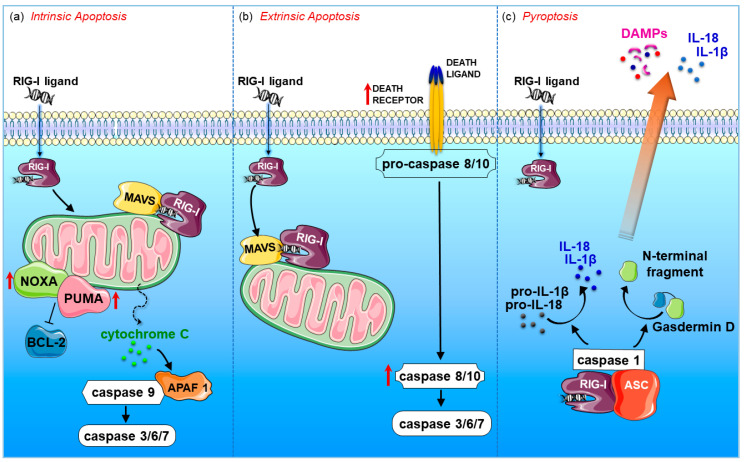
RIG-I activation induces cancer cell death. (**a**) Upon RIG-I activation, expression of the pro-apoptotic genes *NOXA* and *PUMA* is upregulated, followed by cytochrome c release out of mitochondria. Subsequent activation of caspase-9 results in execution of intrinsic apoptotic pathway. (**b**) RIG-I signalling can also induce expression of several pro-apoptotic factors including death receptors that trigger extrinsic apoptotic pathway by activating the caspases cascade. (**c**) Activated RIG-I promotes the recruitment of inflammasome adaptor protein ASC and caspase-1, which drives cleavage of pro-IL-1β and pro-IL-18 and gasdermin D. The N-terminal domain of gasdermin D translocates to the plasma membrane, causing cellular swelling and ultimately pyroptosis. ASC, Apoptosis-associated Speck with a Caspase-recruitment domain; DAMP, danger associated molecular pattern; IL, intereleukin; MAVS, mitochondrial anti-viral signalling.

Cellular mechanisms by which RIG-I triggers programmed cell death include activation of the intrinsic apoptotic pathway [51,54], the extrinsic apoptotic pathway [50,55,56], and pyroptosis [26,57] (Figure 3).

Intrinsic/mitochondrial apoptosis was reported to occur by RIG-I activation in melanoma cells, lung cancer, cervical cancer, myeloid leukaemia, colon and prostate carcinoma [51,54,58,59,60,61]. This apoptotic pathway is regulated by the proapoptotic BCL2 family members (e.g., NOXA and PUMA) and results in permeabilization of the mitochondrial outer membrane, with subsequent leakage of apoptosis-inducing proteins, such as cytochrome c, out of the mitochondria. This, in turn, activates the proapoptotic protein APAF-1, which induces the cleavage of the proenzyme of caspase-9 into the active form. The caspase cascade ultimately results in the proteolysis of intra-cellular proteins and cellular destruction (Figure 3a). The extrinsic tumour cell death was reported to occur in melanoma, prostate and breast cancers in response to RIG-I signalling [50,55,56]. The extrinsic pathway involves transduction of the apoptotic signal after the engagement of a death receptor by its cognate death ligand. The resulting activation of initiator caspase-8 or -10 then directly activates the effector caspases-3, -6, and -7, effectively executing cellular apoptosis (Figure 3b). In this regard, upregulation of TNF-related apoptosis inducing ligand (TRAIL), caspases 8 and 10 was detected in prostate cancer and breast cancer cells [50,55]. RIG-I signalling can also induce pyroptosis, an immunogenic form of gasdermin D-mediated necrosis, in breast cancer cells [50]. Indeed, RIG-I engagement is able to activate the inflammasome by forming a protein complex containing adaptor proteins named apoptosis-associated speck like proteins (ASC) and caspase 1 [62]. The activated caspase 1 then promotes the cleavage of gasdermin D and the processing of pro-inflammatory cytokines interleukin (IL)-1β and IL-18. The N-terminal domain of gasdermin D translocates to the plasma membrane to form membrane pores, causing cellular swelling and lysis, and ultimately pyroptosis. The release of IL-1β, IL-18, and DAMPs into the extracellular environment triggers danger responses in neighbouring cells, amplifying the local inflammatory effects (Figure 3c).

## 4. RIG-I Activation in Cancer and Immune Cells

Host defence response against viruses-infected cells and tumours share intrinsic molecular features. As a consequence, cancer cells can activate a “viral-like” defence signalling leading to engagement of cytosolic RNA sensing pathway [17,63].

The rationale for selective RIG-I activation in cancer cells is therefore to mimic a viral infection. In this way, either a type I IFN-driven immune response associated with an immunogenic form of cancer cell death mainly mediated by DCs, CD8^+^ T, and natural killer (NK) cells [64], or a direct cancer cell apoptosis can be induced [54] (Figure 2). The latter aspect is of particular relevance in poorly immunogenic tumours to mediate in vivo therapeutic effect of RIG-I agonists [50,65,66].

Bifunctional small interfering RNAs with 5′-3P ends have been developed for concomitant silencing of intratumoural oncogenic or immunosuppressive genes and RIG-I activation. Seminal studies were conducted to examine the potential and the efficacy of this combined approach. In a murine melanoma model, a *BCL2*-specific 3p-siRNA, designed to silence the anti-apoptotic *BCL2* gene and stimulated RIG-I-mediated production of type I IFNs, was reported to lead massive apoptosis in lung metastasis. The therapeutic activity also required recruitment and activation of NK cells in lung tissue [58]. Likewise, gene silencing of transforming growth factor (TGF)-β1 was achieved through a 5′-3P-siRNA that simultaneously triggers RIG-I in a murine model of pancreatic cancer. This strategy showed a therapeutic efficacy that was dependent on CD8^+^ T-cells, whereas NK cells were dispensable [67,68]. Such an approach was further validated in other human cancer types. A bifunctional 5′-3P-siRNA combining vascular endothelial growth factor silencing and RIG-I activation inhibited tumour neovascularization, showing a potent antitumour effect in a murine model of lung cancer [60]. Treatment of human drug-resistance leukaemia cells with 5′-3P-siRNA-multi-drug resistance 1 (MDR1) down-regulated MDR1 expression and induced RIG-I-dependent immune and pro-apoptotic effects, suggesting a possible synergistic role for 3p-siRNA-MDR1 in anti-leukaemia therapy [61].

A novel RIG-I-dependent defence pathway, which relies on the vesicle-mediated cross-talk between RIG-I activated cancer cells and immune cells, was recently described in a melanoma mouse model [69]. RIG-I stimulation in melanoma cells induced the release of extracellular vesicles, which expressed enhanced levels of the inducible NKp30 ligand (BAG6) on their surface. The interaction between BAG6 and the NK p30 receptor on NK cells triggered NK cell-mediated lysis of melanoma cells. Systemic administration of melanoma-released vesicles induced a strong antitumour activity in vivo.

Very recently, the study by Dassler-Plenker et al. also demonstrated functional RIG-I expression in naïve human NK cells and their direct activation upon RIG-I ligand (3pRNA) transfection. NK stimulation resulted in increased surface expression of membrane-bound TRAIL, which in turn initiated the extrinsic apoptosis in melanoma cells. TRAIL-dependent killing is induced not only in human allogeneic but also in autologous human leukocyte antigen (HLA) class I-positive and negative melanoma cells [56]. Strikingly, NK cells showed their TRAIL-mediated cytotoxic function only toward cancer cells, sparing healthy normal cells. Non-malignant cells are likely protected by the expression of the decoy receptors TRAIL-R3 and TRAIL-R4, which lack a functional cytoplasmic death domain and fail to induce cell death. Malignant cells are actually highly sensitive to RIG-I-dependent proapoptotic signals. siRNA-mediated *BCL2* silencing as well as TRAIL-dependent NK cells activation provoked massive cell death in cancer cells, while sparing healthy normal cells due to the expression of Bcl-xL, which counteracts RIG-I mediated apoptosis [54], or TRAIL-R3 and TRAIL-R4, which antagonise TRAIL-death signal [56]. Therefore, triggering the immunoreceptor RIG-I in NK cells could offer an improved approach to exploit the anti-tumour effects of membrane-bound TRAIL, although conflicting results have been reported on the relevance of NK cells in RIG-I-based immunotherapy. RIG-I activation has indeed been demonstrated to induce an anti-tumour effect in mouse models that is in part dependent [50,55,58,70] or independent on NK cells [51,67,71,72].

## 5. Evaluation of RIG-I Agonists in Cellular and Preclinical Models of Cancers

Recent understanding of the dual effect of RIG-I activation (i.e., selective tumoricidal activity and activation of immune cells in TME) led to new therapeutic opportunities for cancer immunotherapy. The characteristics of RNA ligands relevant to the design of selective and potent RIG-I agonists are also known [36]. Ligand length, structure, and modifications are essential for activation of RIG-I signalling pathway, and to achieve an appropriate immunostimulatory response.

Some RIG-I agonists showing stability and functional design are being developed and their antitumour efficacy is being currently tested in cellular and animal models of cancers [50,51,66,71,72,73].

By modifying the length, sequence, and structure of the 5′ end of Vesicular Stomatitis Virus RNA, a 99-nucleotide, uridine-rich hairpin 5′-3P-RNA (named M8) was developed [74]. M8 induced in vitro and in vivo a more robust and specific inflammatory response compared to other sequence-modified 5′-3P structures, aptamers and poly(I:C) [74]. Castiello et al. investigated the potential of M8 agonist as anti-cancer agent by analysing its ability to induce apoptosis and activate innate immunity. M8 was effective in inducing an IFN-dependent intrinsic cell death, characterised by the expression of markers of ICD-related DAMPs, as well as immune response in different cancer cell types including metastatic melanoma Mel1007 and Mel120, lung adenocarcinoma A549, colon carcinoma HCT116, and prostate carcinoma PC3 [51]. In addition, RIG-I activation switched on multiple processes within TME. M8-treated melanoma cells stimulated the phagocytic potential of DCs, which increased the expression of HLA and co-stimulatory molecules, finally promoting the inflammatory responses. DCs effectively engulfed apoptotic tumour materials and exhibit enhanced tumour-associated antigens cross-presentation ability to naïve CD8^+^ T-cells, due to the IFN-enriched microenvironment that upregulate surface expression of major histocompatibility complex (MHC) class I molecules [51]. The crucial role of DCs in mediating RIG-I-induced T-cell priming was also demonstrated in other studies [67,71,75,76] (Figure 2).

A recent study reported the development of novel specific and potent synthetic activators of RIG-I receptor [77]. The 5′-3P stem-loop RNAs (SLRs) present a single duplex terminus that fits precisely into the RNA binding pocket of RIG-I, therefore binding only a single RIG-I monomer. The stem-loop design provides resistance to nucleases and enhance structural stability of the complex, a key determinant of RIG-I ligand potency. SLRs are specifically and functionally recognized by RIG-I in human cells [77]. Once introduced into mice, SLR10 and SLR14 (duplex length of 10 and 14 bp, respectively) induced high-levels of type I IFNs and activation of distinct genes essential for antiviral response and innate immunity. Therefore, this study also represented the first in vivo genome-wide assessment of the gene expression profile following RIG-I stimulation [77].

Intratumoural delivery of SLR14 agonist provoked an effective antitumour response in both immunogenic (YMR 1.7) and poorly immunogenic (B16F10) melanoma mouse models by activating the cytosolic RIG-I pathway in different cell populations, i.e., tumour cells and non-tumour cells [66]. The TME was profoundly modified after ligand administration. SLR14 treatment actually induced a significant increase of immune infiltrating leukocytes, including CD11b^+^ myeloid cells, CD8^+^ T-cells and NK cells, while the immunosuppressive CD4^+^ FoxP3^+^ Tregs were reduced. SLR14 was mainly taken up by CD11b^+^ tumour-infiltrating myeloid cells, which were later detected in the draining lymph nodes after the treatment. Cytotoxic CD8^+^T lymphocytes were also present in the draining lymph nodes, where they experienced enhanced tumour-specific Ag cross-priming, thus eliciting a systemic T-cell-mediated immune response (abscopal effect). These data indicate that adaptive immunity was required for tumour clearance as well as to hamper metastatic spreading. Remarkably, antitumour immune response was partially mediated by T-cells in poorly immunogenic melanoma tumour, where SLR14 administration also induced direct activation of RIG-I pathway resulting in tumour cell death [66]. Most of genes associated with the RIG-I pathway, type I IFNs and ISGs, were significantly up-regulated after SLR14 treatment. Furthermore, higher transcriptional level of many cytokines/chemokines and many genes associated with lymphocyte activation and differentiation, and Ag presentation was also reported [66]. This is in line with a previous report showing enhanced induction of antiviral and inflammatory genes in M8-treated primary human DCs, compared to treatment by other RNA agonists [74].

Despite being the second leading cause of cancer death among women, little is known about RIG-I signalling in breast cancer. Previous reports identified RIG-I as belonging to a pro-inflammatory and antimetastatic gene expression signatures in MDA-MB-435 human breast cancer cells [78,79]. Recently, Elion and colleagues assessed the efficacy of a synthetic RIG-I agonist (SLR20) in a breast cancer cell panel representing each of three major clinical subtypes. The engineered SLR20 was able to induce tumour cell killing due to extrinsic apoptosis and pyroptosis activation [50]. Therapeutic benefit was also reported following in vivo delivery of SLR20 agonist toward poorly immunogenic breast cancer. RIG-I activation modulates TME, resulting in active recruitment of TILs, consistent with a more immunogenic TME. In line with this notion, combination treatment with SLR20 and the ICI αPD-L1 resulted in better control of tumour growth than single treatment. RIG-I signalling thus improved the sensitivity to PD-L1 treatment [50]. It is worth mentioning that SLR14 agonist also induced a synergistic antitumour effect when delivered with anti-PD1 against two immunogenic tumours, melanoma YMR 1.7 and MC38 colon cancer. Tumour growth was remarkably inhibited after combination treatment [66].

Novel combinatorial approaches between RIG-I-enhanced vaccine and ICIs are emerging. A protein vaccination strategy that combines engagement of RIG-I via immunostimulatory 3pRNA, antigen and CTLA-4 blockade was recently reported to prevent and treat murine melanoma [71]. The study of Heidegger et al. demonstrated that RIG-I stimulation synergised with checkpoint blockade to potentiate the efficacy of anticancer vaccine and to generate strong antitumour immunity. In this in vivo models, activation of RIG-I augmented DC-mediated cross-presentation of exogenous antigen and subsequent cross-priming of T-lymphocytes. The RIG-I adapter MAVS and DC-intrinsic type I IFN signalling are functionally required for optimal T-cell expansion and cytolytic activity. These findings validate previous studies demonstrating that IFN I signalling is critical for DCs recruitment in the TME, cross-priming of naïve cytotoxic T-cells, and tumour rejection [12,80,81]. In a prophylactic vaccination schedule, CTLA-4 blockade significantly boosted expansion of Ag-specific CD8^+^ T-cells, which translated into strengthened antitumour immunity with systemic reduction of metastatic tumour burden. Importantly, this combined vaccination approach also showed high effectiveness in a therapeutic setting leading to complete tumour regression and long-term survival in all animals [71].

While targeting RIG-I with dsRNA mimetics has shown promising results in the treatment of different solid tumour models, pre-clinical investigation for such a therapeutic approach in haematological malignancies remains less numerous. In a recent publication, the responsiveness of AML to 3P-RNA treatment was assessed in a murine syngeneic model. Administration of 3P-RNA resulted in a specific CD8^+^T-cell-mediated response against disseminated AML and long-term survival in tumour-bearing mice. All surviving mice showed an immunological memory able to protect themselves from a rechallenge with AML cells [72]. The key role of type I IFN signalling for induction of antitumoural immune response was also validated in this model. Interestingly, 3P-RNA treatment using an anti-PD-1 antibody substantiated the notion of a “priming role” of 3P-RNA that sensitize leukemic cells to checkpoint inhibition. This concept has already been described using oncolytic viruses [82] and STING agonists [83] in the same cancer model. 

Recent innovative approaches have suggested exploit RIG-I-mediated apoptosis and convert cancer cells into vaccines. Intrinsic RIG-I activation within melanoma cells by in vitro transfection with 3pRNA resulted in immunogenic cell death, turning melanoma cells into an IFN-I-releasing cellular antitumour vaccine [73]. Immunisation of mice with B16.OVA cells undergoing RIG-I-mediated cell death induced CD8^+^ T-cell cross-priming and protected animals from subsequent melanoma challenge. Adaptive antitumoural response was also evoked after RIG-I activated cellular vaccine in a pre-established melanoma model. These results endorsed the notion that RIG-I receptor activation improves antigen-specific CTL cross-priming [81].

## 6. The RIG-I Signalling Pathway: From Basic Research to Clinical Translation

Several recent clinical trials and some notable earlier clinical studies [84,85,86,87,88,89,90,91,92,93,94,95,96,97,98], based on non-infectious RIG-I activation have reached the attention of pharmaceutical companies in Japan and USA (see Table 1).

These clinical approaches provide a cancer treatment option to induce immunogenic tumour cell death by enhancement of tumour neoantigen presentation, and by stimulation of pro-inflammatory cytokines and cytotoxic TILs activity.

With the recent advancements in immune-based cancer therapies, the delivery of therapeutic agents directly into tumours gains importance as it allows to attack the tumour from within. Intratumoural delivery of RIG-I agonists or mimetics can result in improvements in local tumour immune activation. Notably, local delivery reduces systemic toxicity of cancer treatment while helping to overcome barriers imposed by the inhibitory effects of the TME. Preclinical and early clinical studies have suggested that intratumoural delivery are able to convert a “cold”, non-T-cell inflamed tumour into a “hot”, T-cell inflamed tumour. By successfully administration of immunostimulatory agents into a tumour site, local intratumoural drug delivery hold the potential to drive sustained, systemic immune responses. 

### 6.1. Clinical Trials Based on Targeting of RIG-I with RIG-I Mimetics

Elion and colleagues investigated therapeutic delivery of RIG-I mimetics in preclinical models of estrogen receptor positive (ER^+^) breast cancer. They found that ligand SLR20 treatment led to upregulation of RIG-I expression and inflammatory signals in the tumours. Further, RIG-I activation resulted in increased TILs recruitment in the TME, thereby augmenting the sensitivity to anti-PD-L1-based therapy [50]. These findings supported a clinical study employing SLR20 (MK-4621/RGT100 Merck/Rigontec) (NCT03065023) that evaluated safety and preliminary antitumour activity in patients with advanced solid tumours and lymphomas [26,84,99]. It was reported that the drug formulation appeared to be safe and tolerable for all participants, with no dose-limiting toxicities, up to approximately 2 years of treatment (Table 1).

Another clinical trial (NCT03739138) was recently opened to assess the efficacy of MK4621 as monotherapy and in combination with pembrolizumab, an anti-PD-1, in patients with advanced and recurrent tumours. The synthetic RNA oligonucleotide was intratumorally/intralesionally delivered via the JetPEI™ linear polyethylenimine nucleic acid delivery system. The study, estimated to be completed upon April 2021, includes safety, tolerability, drug pharmacokinetics evaluation and plasma cytokine profiles [85,100] (see Table 1).

A novel adjuvant, termed RNAdjuvant^®^/CV8102, consisting of a single-stranded polyU-repeats rich non-coding RNA complexed with a polymeric carrier formed by disulphide-crosslinked cationic peptide was recently developed [112]. RNAdjuvant^®^ mediates enhancement of an influenza subunit vaccine responses through concurrent Toll-like receptors and RIG-I-like helicase signalling [113]. The efficacy of RNAdjuvant^®^ to enhance antigen-specific CD8^+^ T-cell responses and to mediate anti-tumour responses was demonstrated in the syngeneic TC-1 tumour, a murine model of human papillomavirus (HPV)-induced cervical cancer. RNAdjuvant injection induced local upregulation of inflammatory cytokines, chemokine and type I IFNs and did not induce systemic effects. Therefore, this adjuvant combines a remarkable immunostimulatory activity together with an excellent pre-clinical safety and toxicity profile [112]. Intratumoural CV8102 injection demonstrated anti-tumour activity and synergized with systemic PD-1 inhibition in preclinical models [114]. Such evidence supports clinical translation of this agonist, which is currently underway (NCT03203005, NCT03291002) using RNAdjuvant^®^ from CureVac AG in advanced solid tumours [86,87]. The HEPAVAC-101 (NCT03203005) [86] clinical trial is being carried out by a combination of multi-peptide-based HCC vaccine (IMA970A), and the CV8102 in patients with very early, early, and intermediate stages of HCC [101]. This trial is designed to investigate safety and efficacy of the combination therapy following a single pre-vaccination infusion of low-dose cyclophosphamide. Analysis of immunological effects induced by RNAdjuvant^®^ in patients who underwent chemotherapy demonstrated CD4^+^ T-cell differentiation and production of pro-inflammatory cytokines and chemokines. These findings suggest that RNAdjuvant^®^ holds a great capability of initiating the innate immune response coupled to the ability of recruiting immune cells for potentiating the effector arm of anti-cancer immunity [115].

Phase 1 dose escalation and expansion clinical trial NCT03291002 has been developed to investigate CV8102 as a single agent and in combination with licensed anti-PD-1 antibodies in patients with advanced melanoma, cutaneous squamous cell carcinoma, squamous cell carcinoma of head and neck, or adenoid cystic carcinoma [87]. Intratumoural single agent CV8102 appeared well tolerated and showed preliminary evidence of clinical efficacy with shrinkage of injected and non-injected lesions in a total of 20 patients [103].

### 6.2. Clinical Trials Based on Oncolytic Viruses

Oncolytic viruses are capable of altering the TME immune landscape. These viruses disrupt the immunosuppressive TME and can be used to create a “pro-immune” microenvironment that promotes robust and long-lasting host antitumour immune responses [116].

Hemagglutinating virus of Japan (HVJ)-based oncotherapy has gained attention due to its unique anticancer mechanisms, which are different from those of the conventional oncolytic viruses that kill cancer cells by cancer cell-selective replication. Ultraviolet irradiation of HVJ results in fragmentation of the virus RNA genome into many short RNAs, by losing the virus replication activity while retaining the oncolytic properties of HVJ. UV-irradiated HVJ, which is known as HVJ-envelope (HVJ-E), has intrinsic anti-tumour activities including the activation of multiple anti-tumour immunities and the induction of cancer-selective cell death. Even though HVJ-E-mediated cytotoxic mechanisms differ depending on the cancer cell type, the viral RNA genome fragments are recognized by RIG-I, which causes downstream induction of tumour cell apoptosis [106,117,118]. Very encouraging preclinical results showed that 85% of severe combined immunodeficiency (SCID) mice intratumorally injected with HVJ-E became tumour-free [55]. These proof-of-concept data resulted in numerous clinical studies.

Some currently underway clinical trials are focussed on the evaluation of safety, tolerability, and preliminary efficacy of HVJ-E administration in patients with castration resistant prostate cancer [88,89,91]. In phase 1/2 clinical trials, which started in July 2011 and May 2013 [88,89,90], HVJ-E, termed GEN0101, was repeatedly injected using dose increase design in docetaxel resistant patients or unable to receive this chemotherapy. Fujita and colleagues assessed anti-tumour immunity at days 0, 12, and 28 of each cycle reporting an increased serum anti-HVJ-E antibody titre, whereas IL-6, IFN-α/β, and IFN-γ serum levels, and NK cell activity showed no significative changes [104].

In the single-arm, dose-escalation study (UMIN000017092) [91], GEN0101 was administered four times per two weeks, with two weeks of observation. Each patient received two-cycle treatment period. To determine the appropriate dosing strategy, patients received 30,000 milli neuraminidase unit (mNAU) or 60,000 mNAU per injection with a prostate biopsy performed before and after the HVJ-E injection [119,120]. Despite overall tolerable safety profiles and increased NK cell activity, patients in the high-dose group showed limited anti-tumour effect, lymph node metastasis at baseline, and stable disease. Therefore, no patient showed a decrease of serum prostate-specific antigen (PSA) levels for 8 weeks [105]. It was demonstrated that HVJ-E fuses to prostate cancer cells through GD1a ganglioside, whose different expression among patients is regulated by androgen-dependent promoter demethylation [121]. Therefore, Fujita et al. suggested that an analysis of GD1a expression might help in patient selection and that GEN0101 treatment might be more effective in localized or metastatic hormone-naïve prostate cancer with no local therapy administered to the prostate [104,105]. Administration of HVJ-E into melanoma lesions has been reported as a different intralesional immunotherapy. In a mouse model of melanoma, the combination of chemotherapy with HVJ-E containing therapeutic molecules induced tumour-specific apoptosis and CTL activity [106,107]. A single-arm, phase 1/2a clinical trial (UMIN000002376) was performed as monotherapy in six patients with metastatic melanoma to evaluate safety, antitumour immunity, and clinical effect [92].

Patients were separated into two groups (*n* = 3 each) and received low (3000 mNAU) and high (10,000 mNAU) doses of HVJ-E three times a week, with at least 1 day interval, for a total of two weeks. Investigators reported that treated and non-treated melanoma lesions showed a reduced tumour burden mediated by the antitumour immunity. Vitiligo, which is known to be a prognostic factor and reflects the immune response to immunotherapy in melanoma, was observed around treated and non-treated skin metastasis. Overall, these results suggested the bystander effect caused the HVJ-E administration. The antitumour effects on target lesions are reinforced in a dose-dependent manner considering that complete and partial responses were observed in 33% of low-dose group and in 58% of high-dose group [122].

The recent UMIN000012943 phase 1 clinical study confirmed the tolerability of HVJ-E treatment at higher doses (30,000 and 60,000 mNAU) [93]. This result led to phase 1/2 clinical trials that combines HVJ-E with pembrolizumab to demonstrate the ability of HVJ-E in augmenting the antitumour immunity of ICB [122].

An open dose-escalation clinical trial (UMIN000019345), started on September 2015, will aim to investigate safety, tolerability, and efficacy of GEN0101 administration in patients with chemotherapy-resistant malignant pleural mesothelioma [94]. Investigators will assess the dose limiting toxicity (60,000 mNAU/injection) to determine the recommended dosage for a possible phase 2 study.

The most recent HVJ-E clinical trial combined DCs and tumour fusion cells (HiDCV-OS1 Hybrid cell) with subcutaneous administration of GEN0101 in patients with recalcitrant residual or relapsed ovarian cancer. This study was designed to evaluate the safety and efficacy of this novel immunotherapy (UMIN000031281) [95].

### 6.3. Clinical Trials Based on Epigenetics Drugs

Two separate preclinical studies conducted in ovarian cancer and colorectal cancer cells demonstrated that epigenetic drugs will “trick” cancer cells into behaving as virus-infected cells [123,124]. Pharmacological DNA demethylation actually induces expression of endogenous retroviral transcripts, which in turn fold into RNA secondary structures that can be sensed by the dsRNA sensors. This results in a “viral mimicry” programme in which treated cells mount an innate immune response to the perceived infection by a retrovirus, resulting in antitumour immunity against the cancer cells [123,124,125]. Recent work in patients with myelodysplastic syndromes found that those patients who responded to the DNA methyltransferase inhibitor 5-azacitidine were able to induce inflammatory- and immune-related responses [126]. This provides in vivo clinical validation for the concept that viral mimicry is a key antitumour mechanism induced by DNA demethylating agents.

Three studies employing the 5-azacitidine deserve to be mentioned (see Table 1).

A phase 2 study (NCT01105377) using azacitidine in combination with a histone deacetylase inhibitor, entinostat, was conducted in metastatic colorectal cancer patients [96]. From April 2010 to December 2011, 46 patients enrolled in the study were subcutaneously treated with azacitidine and orally with entinostat, repeating the treatment every 28 days in the absence of serious adverse events. The study failed to meet the primary response evaluation criteria in solid tumours (i.e., RECIST) endpoint of tumour response rate, with a median progression-free survival of 1.9 months and an overall survival of 8.3 months [109]. Similar response was observed in a multicentre phase 2 study of azacitidine and entinostat in women with advanced HER2-negative, hormone-resistant, or triple-negative breast cancers, treated with the same therapeutic protocol [97]. Investigators did not observe any clinical response in triple-negative breast cancer patients, with a median follow-up of 6.6 months. A partial response was observed in the hormone-resistant cohort treated with epigenetic therapy alone, due to the fact that DNA methylation is more abundant in estrogen-receptor-positive tumours than in basal-like tumours, which are usually triple-negative breast cancers [110]. A large phase 2 study evaluating the priming effect of hypomethylating agents on anti-PD1-antibodies is ongoing and recruiting [98]. Moreover, 120 patients with recurrent, metastatic non-small cell lung cancer will be treated with azacitidine and entinostat to sensitize tumours to subsequent therapy with the monoclonal anti-PD1 antibody nivolumab [111].

## 7. Conclusions 

The immunoreceptor RIG-I has emerged as a promising target for development of novel anticancer therapeutics. In the tumour context, the RIG-I signalling drives transcriptional activation of a broad spectrum of pro-inflammatory genes that includes type I IFNs and pro-inflammatory cytokines, followed by immunogenic cell death. Furthermore, RIG-I receptor activation promotes innate immune activation in TME. Thereby, targeting the innate response with RIG-I agonists is a potential strategy to trigger de novo inflammation, Ag-specific DCs cross-presentation, and T-cell cross-priming, and to heightened ICIs sensitivity in tumours, especially in non-T-cell-inflamed types. Several approaches using combination of RIG-I mimetics with ICIs have been explored, mostly in preclinical settings, giving encouraging results.

Currently, there are several ongoing phase 1 and 2 clinical trials, mostly applying RIG-I agonists as monotherapy. However, all of the evidence suggested that combined use of RIG-I agonists with ICIs or with cytotoxic chemotherapeutics may provide favourable treatment benefit.

Despite the potential widespread applicability for therapeutic RIG-I agonists, a number of caveats concerning the innate immune activation has to be considered. In an immunosuppressive TME, a crucial event is the presence of immature, tolerogenic DCs [127]. In this context, RIG-I activation could induce tolerogenic DCs to produce IL-10. Besides hampering activation of CD8^+^ T-cells, IL-10-releasing tolerogenic DCs elicit the differentiation of regulatory T-lymphocytes [128]. This may lead to positive reinforcement of intratumoural IL-10-dependent tolerance.

In addition to possible “on target” (i.e., unrestrained inflammation) and “off target” effects of RIG-I activation, effective delivery of immune stimulators is a major barrier to the employment of synthetic RIG-I agonists in the immunotherapy. The JetPEI vehicle was used to facilitate in vivo delivery of RIG-I ligands [66,71]. Both subcutaneous and intravenously administration resulted in MAVS-dependent expansion of specific CD8^+^ T-cells in local draining lymph nodes and the spleen, although the cytolytic activity was less efficient following the intravenous application route [71]. Intratumoural delivery should provide additional benefits through RIG-I-mediated apoptosis in cancer cells as well as local recruitment of immune cells in the TME. This may lead to a significant antitumour response. In vivo delivery of SLR mimetics has been recently achieved through nanoparticle-mediated delivery, which may be another potential therapeutic approach to be considered. It is worth noting the nanoparticle-mediated delivery of the RIG-I agonist induced tumour cell killing in breast cancer and modulated TME towards a favourable therapeutic outcome [50]. Thus, the development of efficacious delivery systems with site-specificity (e.g., draining lymph nodes) will be needed for improvement of immune response to RIG-I agonists.

It is anticipated that cytosolic RNA sensing activation combined with other immunotherapies might be employed in the future to boost the immune response against cancer.

## Figures and Tables

**Table 1:** Representative clinical trials in oncology using RIG-I signalling.

Therapy and Diseases	Status	Phase	Enrolment	Trial and Compound	CT Identifier and Drug	Sponsor	Start Date/Last Update	References
Study of intralesional administration of MK-4621 (RGT100) in adult participants w/advanced or recurrent tumours (MK-4621-001/RGT100-001)	Terminated	Phase 1/Phase 2	15	MK-4621/RGT100 (MK-4621-001/RGT100-001)	NCT03065023 A synthetic RNA oligonucleotide	Rigontec GMBH Merck Sharp & Dohme Corp.	April 2017/July 2019	[26,84,99]
Intratumoral/intralesional administration of MK-4621/JetPEI™ w/or w/o pembrolizumab in participants w/advanced/metastatic or recurrent solid tumours (MK-4621-002)	Active, not recruiting	Phase 1	72	MK-4621/JetPEI™ Pembrolizumab	NCT03739138 A synthetic RNA oligonucleotide as monotherapy and in combination with anti-PD-1 via the JetPEI™	Merck Sharp & Dohme Corp.	December 2018/April 2020	[85,100]
IMA970A plus V8102 in very early, early and intermediate stage hepatocellular carcinoma patients	Completed	Phase 1/Phase 2	22	IMA970A CV8102 cyclophosphamide	NCT03203005 Liver cancer peptide vaccine plus RNAdjuvant^®^ and cyclophosphamide	National Oncological Institute “Pascale Foundation” (HepaVAC Foundation) CureVac AG	September 2018/February 2020	[86,101,102]
Study of intratumoral CV8102 w/or w/o standard of care anti-PD-1 therapy in cMEL, cSCC, hnSCC, and ACC	Recruiting	Phase 1	233	CV8102 Standard anti-PD-1 therapy	NCT03291002 RNAdjuvant^®^ as monotherapy and in combination with anti-PD-1 Abs	CureVac AG	September 2017/April 2020	[87,103]
Safety and efficacy study of intratumoral and subcutaneous injection of HVJ-E to castration resistant prostate cancer patients	Recruiting	Phase 1/Phase 2	12	GEN0101	UMIN000006142 Inactivated HVJ-E	Osaka Hospital GenomIdea Inc.	July 2011/December 2014	[88,104]
Study of subsequent injection of HVJ-E to castration resistant prostate cancer patients enrolled in the UMIN000006142 clinical trial	Enrolling by invitation	Phase 1/Phase 2	9	GEN0101	UMIN000010840/NCT02502994 Inactivated HVJ-E	Osaka Hospital GenomIdea Inc.	May 2013/June 2013	[89,90]
Dose-escalation, safety/tolerability and preliminary efficacy study of intratumoral and subcutaneous administration of GEN0101 in patients with recurrence of CRPC	Follow-up complete	Phase 1	12	GEN0101	UMIN000017092 Inactivated HVJ-E	Osaka Hospital GenomIdea Inc.	March 2015/November 2017	[91,105]
Administration of inactivated HVJ-E for advanced malignant melanoma patients (stage IIIC or stage IV)	phase I completed	Phase 1/Phase 2	6	GEN0101	UMIN000002376 Inactivated HVJ-E	Osaka University Keio University	July 2009/January 2012	[92,106,107]
Dose-escalation, safety/tolerability and preliminary efficacy study of intratumoral administration of GEN0101 in patients with advanced melanoma.	Completed	Phase 1	15	GEN0101	UMIN000012943 Inactivated HVJ-E	Osaka University Hospital Japan Agency for Medical Research and Development	December 2013/January 2017	[93]
Dose-escalation, safety/tolerability and preliminary efficacy study of intratumoral and subcutaneous administration of GEN0101 in patients with chemotherapy-resistant malignant pleural mesothelioma	Recruiting	Phase 1	12	GEN0101	UMIN000019345 Inactivated HVJ-E	Osaka University GenomIdea Inc	September 2015/October 2015	[94]
Safety and preliminary efficacy study of HiDCV-OS1 and GEN0101 in patients with chemotherapy-resistant ovarian cancer	Enrolling by invitation	Phase 1	6	GEN0101 HiDCV-OS1	UMIN000031281 Inactivated HVJ-E and dendritic/tumor fusion cells	Osaka University	January 2018/May 2018	[95]
Study of azacitadine and entinostat in patients with metastatic colorectal cancer	Completed	Phase 2	47	Azacitidine/entinostat	NCT01105377 DNA methyltransferase inhibitor and DNA methyltransferase and HDAC inhibitor	National Cancer Institute (NCI)	April 2010/August 2014	[96,108,109]
Azacitidine and entinostat in treating patients with advanced breast cancer, triple-negative and hormone-refractory	Active, not recruiting	Phase 2	58	Azacitidine/entinostat	NCT01349959 DNA methyltransferase inhibitor and HDAC inhibitor	National Cancer Institute (NCI)	April 2011/March 2020	[97,108,110]
Study of epigenetic therapy with azacitidine and entinostat with Concurrent nivolumab in subjects with recurrent metastatic non-small cell lung cancer.	Recruiting	Phase 2	120	Azacitidine/entinostat/nivolumab	NCT01928576 DNA methyltransferase inhibitor and HDAC inhibitor and monoclonal anti PD-1 protein antibody	Sidney Kimmel Comprehensive Cancer Centre at Johns Hopkins	August 2013/April 2020	[98,111]

Abbreviations: RIG-I, retinoic acid-inducible gene I; PEI, polyethylenimine; anti-PD-1, anti-programmed cell death; TLR, toll-like receptor; cMEL, cutaneous melanoma; cSCC, cutaneous squamous cell carcinoma; hnSCC, head and neck squamous cell carcinoma; ACC, adenoid cystic carcinoma; HVJ-E, hemagglutinating virus of Japan Envelope; CRPC, castrate-resistant prostate cancer; HDAC, histone deacetylases.
